# Supplementary material for: Impact of the Cooperative Health Insurance System in Saudi Arabia on Universal Health Coverage—A Systematic Literature Review
Source: Healthcare (Basel). 2025 Jan 1;13(1):60. doi: 10.3390/healthcare13010060 (PMC11719570; doi:10.3390/healthcare13010060)
Supplement: Supplementary file 1 [file healthcare-13-00060-s001.zip › Supplementary File S1.pdf]

Section and Topic	Item #	Checklist item	Location where item is reported
**TITLE**			
Title	1	Identify the report as a systematic review.	Page 1, Lines 1-4.
**ABSTRACT**			
Abstract	2	See the PRISMA 2020 for Abstracts checklist.	Page 1, Lines 21-22.
**INTRODUCTION**			
Rationale	3	Describe the rationale for the review in the context of existing knowledge.	Page 2, Lines 39-58 and Lines 67-75.
Objectives	4	Provide an explicit statement of the objective(s) or question(s) the review addresses.	Page 2, Lines 58-61.
**METHODS**			
Eligibility criteria	5	Specify the inclusion and exclusion criteria for the review and how studies were grouped for the syntheses.	Page 3, Lines 96-98 and Lines 118-124. Table 1.
Information sources	6	Specify all databases, registers, websites, organisations, reference lists and other sources searched or consulted to identify studies. Specify the datewhen each source was last searched or consulted.	Page 1, Lines 22-23 and Page 2, Lines 82-85.
Search strategy	7	Present the full search strategies for all databases, registers, and websites, including any filters and limits used.	Page 2, Lines 87-94.
Selection process	8	Specify the methods used to decide whether a study met the inclusion criteria of the review, including how many reviewers screened each record andeach report retrieved, whether they worked independently, and, if applicable, details of automation tools used in the process.	Page 3, Lines 100-109.
Data collection process	9	Specify the methods used to collect data from reports, including how many reviewers collected data from each report, whether they worked independently, any processes for obtaining or confirming data from study investigators, and if applicable, details of automation tools used in the process.	Page 3, Lines 110-115.
Data items	10a	List and define all outcomes for which data were sought. Specify whether all results that were compatible with each outcome domain in each study were sought (e.g., for all measures, time points, analyses), and if not, the methods used to decide which results to collect.	Page 3, Lines 118-124.
	10b	List and define all other variables for which data were sought (e.g., participant and intervention characteristics, funding sources). Describe any assumptions made about any missing or unclear information.	Page 3, Lines 110-115.
Study risk of bias assessment	11	Specify the methods used to assess the risk of bias in the included studies, including details of the tool(s) used, how many reviewers assessed each study, whether they worked independently, and, if applicable, details of automation tools used in the process.	Page 3, Lines 121-123
Effect measures	12	Specify the effect measure(s) (e.g., risk ratio and mean difference) used in the synthesis or presentation of results for each outcome.	n.a., this is not a meta-analysis
Synthesis methods	13a	Describe the processes used to decide which studies were eligible for each synthesis (e.g., tabulating the study intervention characteristics and comparing against the planned groups for each synthesis (item #5)).	n.a., this is not a meta-analysis
	13b	Describe any methods required to prepare the data for presentation or synthesis, such as handling missing summary statistics or data conversions.	n.a., this is not a meta-analysis
	13c	Describe any methods used to tabulate or visually display the results of individual studies and syntheses.	n.a., this is not a meta-analysis
	13d	Describe any methods used to synthesise results and provide a rationale for the choice(s). If meta-analysis was performed, describe the model(s), method(s) to identify the presence and extent of statistical heterogeneity, and software package(s) used.	n.a., this is not a meta-analysis
	13e	Describe any methods used to explore possible causes of heterogeneity among study results (e.g., subgroup analysis, meta-regression).	n.a., this is not a meta-analysis
	13f	Describe any sensitivity analyses conducted to assess the robustness of the synthesised results.	n.a., this is not a meta-analysis
Reporting bias assessment	14	Describe any methods used to assess the risk of bias due to missing results in a synthesis (arising from reporting biases).	n.a., this is not a meta-analysis
Certainty assessment	15	Describe any methods used to assess certainty (or confidence) in the body of evidence for an outcome.	n.a., this is not a meta-analysis

Section and Topic	Item #	Checklist item	Location where item is reported
Study selection	16a	Describe the results of the search and selection process, from the number of records identified in the search to the number of studies included in the review, ideally using a flow diagram.	Page 4, Lines 128-132. Figure 2.
	16b	Cite studies that might appear to meet the inclusion criteria but which were excluded and explain why they were excluded.	Page 4, Lines 130-132 and Page 5, Lines 140-142. Figure 2.
Study characteristics	17	Cite each included study and present its characteristics.	Page 4-5, Lines 134-148. Table 2.
Risk of bias in studies	18	Present assessments of risk of bias for each included study.	n.a., this is not a meta-analysis
Results of individual studies	19	For all outcomes, present, for each study: (a) summary statistics for each group (where appropriate) and (b) an effect estimate and its precision (e.g. confidence/credible interval), ideally using structured tables or plots.	n.a., this is not a meta-analysis
Results of syntheses	20a	For each synthesis, briefly summarise the characteristics and risk of bias among contributing studies.	n.a., this is not a meta-analysis
	20b	Present results of all statistical syntheses conducted. If meta-analysis was done, present for each the summary estimate and its precision (e.g. confidence/credible interval) and measures of statistical heterogeneity. If comparing groups, describe the direction of the effect.	n.a., this is not a meta-analysis
	20c	Present results of all investigations of possible causes of heterogeneity among study results.	n.a., this is not a meta-analysis
	20d	Present results of all sensitivity analyses conducted to assess the robustness of the synthesised results.	n.a., this is not a meta-analysis
Reporting biases	21	Present assessments of risk of bias due to missing results (arising from reporting biases) for each synthesis assessed.	n.a., this is not a meta-analysis
Certainty of evidence	22	Present assessments of certainty (or confidence) in the body of evidence for each outcome assessed.	n.a., this is not a meta-analysis
**DISCUSSION**			
Discussion	23a	Provide a general interpretation of the results in the context of other evidence.	Page 8-9, Lines 300-334.
	23b	Discuss any limitations of the evidence included in the review.	Page 9, Lines 336-345.
	23c	Discuss any limitations of the review processes used.	n.a.
	23d	Discuss the implications of the results for practice, policy, and future research.	Page 9, Lines 347-357.
**OTHER INFORMATION**			
Registration and protocol	24a	Provide registration information for the review, including the register name and registration number, or state that the review was not registered.	Page 2, Line 84-86.
	24b	Indicate where the review protocol can be accessed or state that a protocol was not prepared.	Page 2, Lines 84-86.
	24c	Describe and explain any amendments to information provided at registration or in the protocol.	n.a.
Support	25	Describe sources of financial or non-financial support for the review and the role of the funders or sponsors in the review.	Page 10, Lines 369.
Competing interests	26	Declare any competing interests of review authors.	Page 10, Lines 374.

Section and Topic	Item #	Checklist item	Location where item is reported
Availability of data, code and other materials	27	Report which of the following are publicly available and where they can be found: template data collection forms; data extracted from included studies; data used for all analyses; analytic code; any other materials used in the review.	Page 10, Lines 372-373.

From: Page MJ, McKenzie JE, Bossuyt PM, Boutron I, Hoffmann TC, Mulrow CD, et al. The PRISMA 2020 statement: an updated guideline for reporting systematic reviews. BMJ 2021;372:n71. doi: 10.1136/bmj.n71
For more information, visit: http://www.prisma-statement.org/
