# Supplementary material for: Impact of the Cooperative Health Insurance System in Saudi Arabia on Universal Health Coverage—A Systematic Literature Review
Source: Healthcare (Basel). 2025 Jan 1;13(1):60. doi: 10.3390/healthcare13010060 (PMC11719570; doi:10.3390/healthcare13010060)
Supplement: Supplementary file 1 [file healthcare-13-00060-s001.zip › Supplementary File S2.pdf]

Table S1: Results of CASP Qualitative Checklist - Total Points

Author, Year	Statement ofaims	Qualitative methodology appropriate	Research design appropriate	Recruitmentstrategy appropriate	Data collection appropriate	The relationship between the researcherand participants considered	Ethical issues considered	Data analysis rigorous	Statement offindings	Is research valuable	The outcome of the checklist(Yes/ Can't tell / No) N (%)
4. Khaliq, 2012	Yes	Yes	Yes	Can't Tell	Yes	Can't Tell	Yes	Can't Tell	Yes	Yes	7 / 0 / 3 (70%)
5. Al-Sharqi & Abdullah, 2013	Yes	Yes	Yes	Can't Tell	Yes	Can't Tell	Yes	Can't Tell	Yes	Yes	7 / 0 / 3 (70%)
15. Al-Hanawi et al., 2017	Yes	Yes	Yes	Yes	Yes	Can't Tell	Yes	Yes	Yes	Yes	9 / 0 / 1 (90%)
17. Rahman & Alsharqi, 2019	Yes	Yes	Yes	Can't Tell	Yes	Can't Tell	Yes	Can't Tell	Yes	Yes	7 / 0 / 3 (70%)
21. Alonazi, 2020	Yes	Yes	Yes	Can't Tell	Yes	Can't Tell	Yes	Yes	Yes	Yes	8 / 0 / 2 (80%)
26. Alasiri & Mohammed, 2022	Yes	Yes	Yes	Can't Tell	Can't Tell	Can't Tell	Yes	Can't Tell	Yes	Yes	6 / 0 / 4 (60%)
30. Alharbi & Nair, 2022	Yes	Yes	Yes	Can't Tell	Yes	Can't Tell	Yes	Yes	Yes	Yes	8 / 0 / 2 (80%)

Table S2: Results of CASP Quantitative Checklist - Total Points

Author, Year	Focused issue addressed	Acceptable recruitment	Exposure measured to minimise bias	Outcome measured to minimise bias	Confounding factors identified	Design/ analysis considers confounding variables	Follow up ofsubjects complete and long enough	Results believed	Can results beapplied to local population	Results fitwith other evidence	Outcome of checklist (Yes/Can't tell / No)
1. Bin Saeed, 1998	Yes	Yes	Yes	Yes	Can't Tell	Can't Tell	No	Yes	Yes	Yes	7 / 2 / 1 (70%)
2. Alnaif, 2006	Yes	Can't Tell	Yes	No	No	No	No	Yes	Yes	Yes	5 / 1 / 4 (50%)
3. Ansari, 2011	Yes	Yes	No	No	No	No	No	Yes	Yes	Yes	5 / 0 / 5 (50%)
6. Bawazir et al., 2012	Yes	Yes	No	Yes	No	No	Yes	Yes	Yes	Yes	7 / 0 / 3 (55%)
7. Alkhamis et al., 2013	Yes	Yes	Yes	Yes	No	No	Yes	Yes	Yes	Yes	8 / 0 / 2 (80%)

8. Bah et al., 2015	Yes	Can't Tell	No	No	No	No	Yes	Yes	Yes	Yes	5 / 1 / 4 (50%)
9. Aldosari et al., 2016	Yes	Yes	Yes	Yes	No	No	Can't Tell	Yes	Yes	Yes	7 / 1 / 2 (70%)
10. Alsubaie et al., 2016	Yes	Yes	Yes	Yes	Yes	Yes	Can't Tell	Yes	Yes	Yes	9 / 1 / 0 (90%)
11. Aldosari et al., 2016	Yes	Yes	Yes	Yes	No	No	Yes	Yes	Yes	Yes	8 / 0 / 2 (80%)
12. Alkhamis et al., 2017	Yes	Yes	Yes	Yes	Yes	Yes	Yes	Yes	Yes	Yes	10 / 0 / 0 (100%)
13. Alkhamis, 2017	Yes	Yes	Yes	Yes	Yes	Yes	Yes	Yes	Yes	Yes	10 / 0 / 0 (100%)
14. Alkhamis, 2018	Yes	Yes	Yes	Yes	Yes	Yes	Yes	Yes	Yes	Yes	10 / 0 / 0 (100%)
16. Al-Hanawi et al., 2018	Yes	Yes	Yes	Yes	Yes	Yes	Yes	Yes	Yes	Yes	10 / 0 / 0 (100%)
18. Ahmed & Al Amri, 2020	Yes	Can't Tell	No	No	No	No	Can't Tell	Yes	Yes	Yes	4 / 2 / 4 (40%)
19. Al-Hanawi et al., 2020	Yes	Yes	Yes	Yes	Yes	Yes	Yes	Yes	Yes	Yes	10 / 0 / 0 (100%)
20. Alkodaymi et al., 2020	Yes	Yes	Can't Tell	Can't Tell	No	No	Yes	Yes	No	Yes	5 / 2 / 3 (50%)
22. Al-Hanawi et al., 2020	Yes	Yes	Yes	Yes	Yes	Yes	Yes	Yes	Yes	Yes	10 / 0 / 0 (100%)
23. Al-Hanawi et al., 2021	Yes	Yes	Yes	Yes	Yes	Yes	Yes	Yes	Yes	Yes	10 / 0 / 0 (100%)
24. Al-Hanawi, 2021	Yes	Yes	Yes	Yes	Yes	Yes	Yes	Yes	Yes	Yes	10 / 0 / 0 (100%)
25. Alharbi, 2021	Yes	Yes	Yes	Yes	Yes	Yes	No	Yes	Yes	Yes	9 / 0 / 1 (90%)
27. Alharbi, 2022	Yes	Yes	Yes	Yes	Yes	Yes	Yes	Yes	Yes	Yes	10 / 0 / 0 (100%)
28. Almalki et al., 2022	Yes	Yes	Yes	Yes	Yes	Yes	Yes	Yes	Yes	Yes	10 / 0 / 0 (100%)
29. Alharbi, 2021	Yes	Can't Tell	Yes	Yes	Yes	Yes	Yes	Yes	Yes	Yes	9 / 1 / 0 (90%)
